# Unraveling morphological brain network disparities Parkinsonian tremor from essential tremor: an artificial intelligence approach for clinical differentiation

**DOI:** 10.1038/s41531-025-01107-8

**Published:** 2025-08-22

**Authors:** Moxuan Zhang, Siyu Zhou, Huizhi Wang, Pengda Yang, Jinli Ding, Xiaobo Wang, Xuzhu Chen, Chaonan Zhang, Anni Wang, Yuan Gao, Qiang Liu, Yueping Li, Tianqi Xu, Zeyu Ma, Yin Jiang, Lin Shi, Chunlei Han, Yuchen Ji, Guoen Cai, Tao Feng, Jianguo Zhang, Fangang Meng

**Affiliations:** 1 Beijing Neurosurgical Institute, Capital Medical University, Beijing, China; 2 Department of Neurosurgery, Beijing Tiantan Hospital, Capital Medical University, Beijing, China; 3 Department of Radiology, Beijing Tiantan Hospital, Capital Medical University, Beijing, China; 4 Department of Neurology, Fujian Medical University Union Hospital, Fujian, China; 5 Department of Neurosurgery, The First Affiliated Hospital of Zhengzhou University, Zhengzhou, Henan China; 6 Center for movement disorders, Department of Neurology, Beijing Tiantan Hospital, Capital Medical University, Beijing, China

**Keywords:** Movement disorders, Parkinson's disease

## Abstract

Tremor-dominant Parkinson’s disease (TD) and Essential Tremor (ET) are the two most common types of tremors, posing huge challenges in diagnosis. This study was to investigate the pathogenesis of tremors using brain morphology and employ artificial intelligence techniques for distinguishing them. The cortical thickness differences in TD were primarily centered on the right precuneus, while in ET were mainly observed in the right medial orbitofrontal cortex. Subcortical analysis revealed that TD patients primarily exhibited an increase in pallidum, whereas ET patients showed a significant reduction in thalamus. Causal network analysis indicated that in TD, the right temporal lobe exhibited the highest out-degree, and gradually extended to motor control regions. In contrast, ET primarily exhibits initial changes in the prefrontal and occipital visual cortices. Finally, by incorporating these specific characteristics, we developed a machine learning model capable of accurately distinguishing between different tremor types, providing valuable insights for clinical practice.

## Introduction

Tremor is defined as an involuntary, rhythmic, and oscillatory movement in a part of the body. While it frequently affects the limbs, it can also involve other regions such as the head, chin, voice, or soft palate, leading to significant disruptions in daily activities^1^. As one of the most common movement disorders, tremor can result from various etiologies, including Parkinson’s Disease (PD), Essential Tremor (ET), dystonic tremor, drug-induced tremor, and so on. Among these, PD and ET represent the two most prevalent types of pathological tremor on a global scale, while other forms remain relatively uncommon^2^.

PD tremor typically manifests as a rest tremor, characterized by rhythmic movements that occur when the affected limb is at rest and diminish during voluntary motion. It is generally low-frequency (4–6 Hz) and is often accompanied by other motor symptoms, including bradykinesia, rigidity, and postural instability^3^. It can also occur some accompanying symptoms, such as constipation and decreased sense of smell^4^. ET is most characterized by postural or action tremor, which is more prominent during specific postures or voluntary movement, with a higher frequency range of 8–12 Hz^5^. It commonly involves bilateral hand tremor and extra-limb regions such as the head and voice, and it often shows a familial inheritance pattern^6^. Despite these distinctions, overlapping features between PD tremor and ET pose significant diagnostic challenges. For instance, with disease progression, ET tremor amplitude can increase, mimicking PD tremor, making it increasingly challenging to differentiate them^7,8^. Furthermore, atypical presentations, such as the coexistence of rest and action tremors or features of dystonic and drug-induced tremors, often blur clinical boundaries, making accurate diagnosis even more difficult^9,10^.

With advancements in MRI technology, the combination of imaging and morphological analysis has provided critical insights into functional brain disorders. Prior studies have identified differences in cortical thickness and gray matter volume in key brain regions, including the frontal, parietal, and temporal lobes, as well as subcortical structures like the thalamus and putamen in ET^11,12^. However, changes in structural connectivity and the evolution of brain networks during the progression of those tremor disease remain underexplored. Recent research suggests that ET and PD may share similar or overlapping mechanisms underlying tremor generation^13^. From a network perspective, this approach may provide valuable insights into the dysfunction in tremor disorders and help identify the common neurological pathways underlying these conditions. Causal Structural Covariance Network (CaSCN) analysis, a recently developed brain network analysis technique based on structural MRI data, has been introduced to explore causal relationships underlying gray matter changes across brain regions^14^. This approach uses granger causality analysis (GCA) to infer the directed influence between brain regions by constructing a pseudo-time series ordered by disease duration. GCA is a widely used technique for mapping directional information flow in the brain by determining whether neural activity in one region precedes and can predict activity in another region^15^. It allows for the assessment of structural progression patterns during disease development and has been successfully applied in a variety of conditions, including movement disorders and psychiatric illnesses^16^. Xu et al. demonstrated that gray matter abnormalities in blepharospasm originated in the right supplementary motor area and gradually spread to the cortico-basal ganglia motor circuit and visual-motor integration pathway^17^. Another study on spinocerebellar ataxia type 3 revealed that structural abnormalities originated in the cerebellar vermis and subsequently propagated along the cerebellum, neostriatum, and further into frontal and parietal cortices^18^. Furthermore, integrating brain network differences across patients through advanced techniques such as machine learning and deep learning has shown great potential for improving diagnostic accuracy^19^.

In this study, we selected tremor-dominant PD (TD) patients as representatives of Parkinsonian tremor and utilized structural and functional MRI to analyze morphological changes in TD patients compared to those with ET. By examining alterations in structural imaging, we identified significant regions of interest (ROIs) as seed points for further CaSCN analysis. This study revealed different disease progression networks in TD and ET patients from the perspective of brain morphology. Finally, based on these unique features, a machine learning model was developed to differentiate between the two types of tremors, offering valuable support for clinical applications.

## Results

### Demographics and clinical characteristics

The final cohort included 69 TD patients and 71 ET patients. No significant differences were found between the groups in terms of age, gender, years of education, and Mini-Mental State Examination (MMSE) scores. TD patients had a later age of onset (54.61 ± 9.02 vs. 49.05 ± 12.84), higher Montreal Cognitive Assessment (MoCA) scores (23.36 ± 3.04 vs. 21.38 ± 4.88), and higher the Hamilton Rating Scale for Anxiety (HAM-A), the Hamilton Rating Scale for Depression (HAM-D), and the Voice Handicap Index (VHI) scores (15.42 ± 9.20 vs. 10.42 ± 7.60, 16.59 ± 9.91 vs. 11.35 ± 7.41, 25.51 ± 22.09 vs. 7.62 ± 18.73), but a shorter disease duration (9.28 ± 7.16 vs. 16.41 ± 12.48). A total of 80 age- and education-matched healthy controls (HC) were recruited for the study. Demographic and clinical characteristics of the TD, ET, and HC groups are summarized in Table 1.

**Table 1:** Patients and healthy controls clinical characteristics

Characteristics	TD subjects (*n* = 69)	ET patients (*n* = 71)	HC subjects (*n* = 80)	*P* value
Age (year)	63.62 ± 6.66	65.31 ± 7.91	63.84 ± 5.16	0.271^a^
Gender (Male/Female)	37/32	32/39	37/43	0.545^b^
Education (year)	14.13 ± 10.51	13.61 ± 4.66	12.98 ± 5.30	0.381^c^
Age at onset (year)	54.61 ± 9.02	49.05 ± 12.84	-	0.007^d**^
Duration (year)	9.28 ± 7.16	16.41 ± 12.48	-	<0.001^d***^
TETRAS	-	30.43 ± 11.14	-	-
FTMT	-	49.62 ± 27.63	-	-
UPDRS-III (med off)	45.75 ± 15.25	-	-	-
UPDRS-III (med on)	22.85 ± 12.91	-	-	-
MMSE	27.57 ± 1.46	26.96 ± 2.36	-	0.102^d^
MoCA	23.36 ± 3.04	21.38 ± 4.88	-	0.005^d**^
HAM-A	15.42 ± 9.20	10.42 ± 7.60	-	0.001^d**^
HAM-D	16.59 ± 9.91	11.35 ± 7.41	-	0.001^d**^
VHI	25.51 ± 22.09	7.62 ± 18.73	-	<0.001^d***^

Values are presents as the mean ± standard deviation.

*TD* Tremor-dominant Parkinson’s disease, *ET* Essential tremor, HC healthy controls, *TETRAS* The Essential Tremor Rating Assessment Scale, *FTMT* Fahn Tolosa Marin Tremor Rating Scale, *UPDRS-III* the Unified Parkinson’s Disease Rating Scale part 3, *MMSE* Mini- Mental State Examination, *MoCA* Montreal Cognitive Assessment, *HAM-A* Hamilton Rating Scale for Anxiety, *HAM-D* Hamilton Rating Scale for Depression, *VHI* Voice Handicap Index.

^a^Kruskal-Wallis test.

^b^Chi-Squared test.

^c^ANOVA test.

^d^Two-sample t-test for ET and TD group.

**p < 0.01, ***p < 0.001.

### Overall cortical thickness alterations in TD and ET patients

**Fig. 1:**
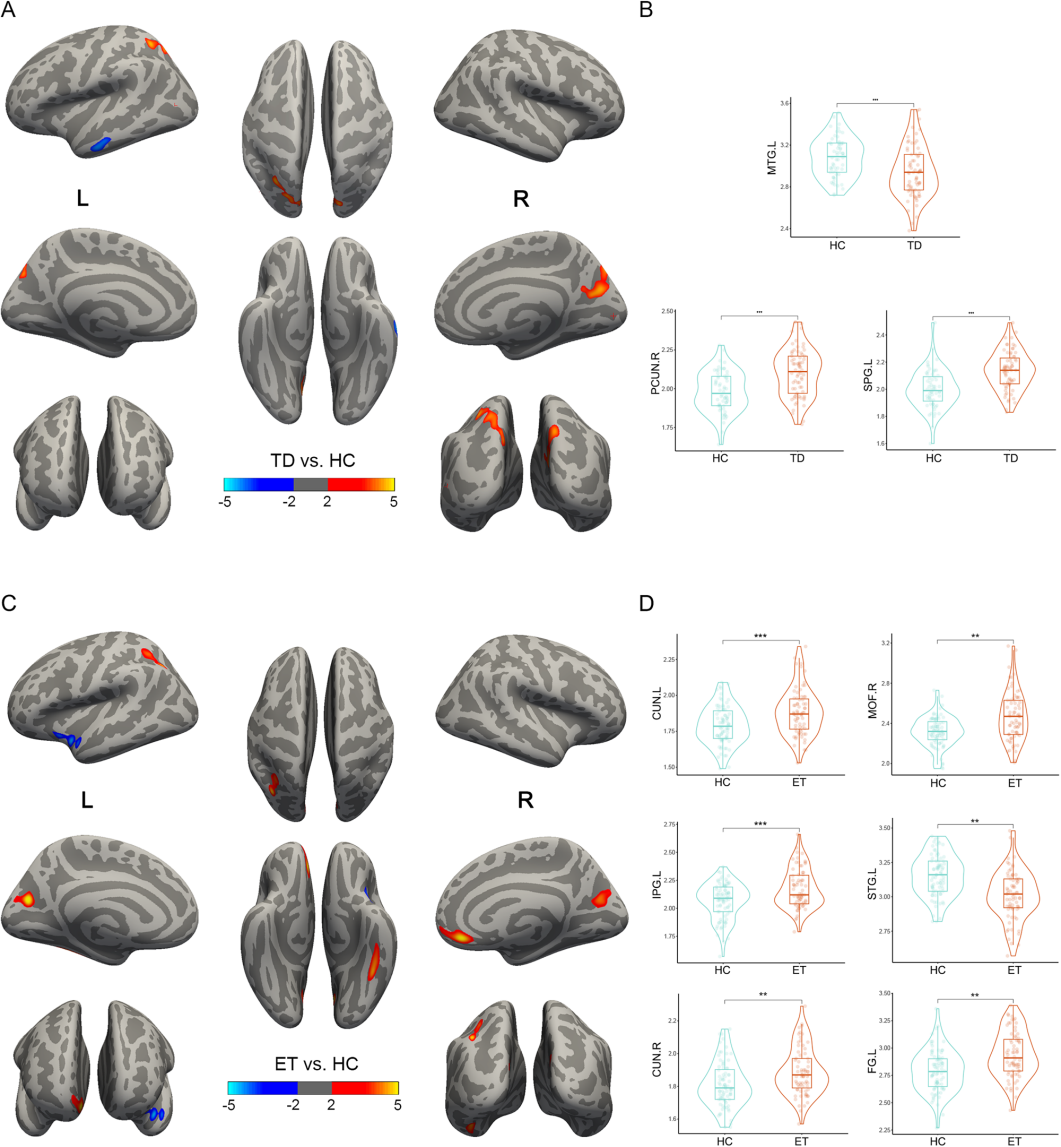
The alteration of cortical thickness in TD and ET patients compared to HC. **A**, **C** Brain regions showing significant cortical thickness alteration in the two patients groups compared to HC, visualized on inflated brain by Freesurfer. Red areas represent an increase in cortical thickness, and the blue areas represent the cortical thickness decreased. **B**, **D** Violin plots directly illustrate the difference in cortical thickness between groups. The results were corrected by MCS with *P* < 0.05. **P < 0.01, ***P < 0.001. TD Tremor-dominant Parkinson’s disease, ET essential tremor, HC healthy controls, MCS Monte Carlo Simulation.

Compared to HC, TD patients exhibited a significant decline in the left middle temporal (MTG.L) but increased in the left superior parietal (SPG.L) and right precuneus (PCUN.R) (Fig. 1A). Cortical thickness differences were shown in Fig. 1B and Supplementary Table 1. In ET patients compared with HC, result showed that the cortical thickness increased in widespread cortical areas (Fig. 1C), including right medial orbitofrontal (MOF.R), left and right cuneus (CUN.L and CUN.R), left inferior parietal (IPG.L), and left fusiform (FG.L), but the left superior temporal (STG.L) decreased. Cortical thickness differences were shown in Fig. 1D and Supplementary Table 2.

In comparisons of early-stage patients, the early-stage TD patients exhibited a significant increase in PCUN.R and right postcentral gyrus (PostCG.R) compared to HC (Supplementary Fig. 1A). In early-stage ET patients, cortical thickening was identified in the MOF.R, right superior temporal gyrus (STG.R), and the SPG.L (Supplementary Fig. 1B). No cortical decreased was found in either early TD or early ET subgroups. These findings suggested that specific cortical alterations were already present at early disease stages, which could facilitate early differential diagnosis.

### Subcortical volume analysis

Three-group ANOVA analysis revealed significant volumetric alterations among the three groups in several regions, including the pallidum, thalamus, cerebellar cortex, ventral diencephalon (Ventral DC), and brainstem. Among these, the pallidum and thalamus exhibited the most significant changes in the nuclei analysis, with bilateral alterations observed. Specifically, post hoc analysis showed that TD patients mainly increased pallidum volume compared with the other two groups (Table 2). In ET patients, there was a significant decrease in the thalamus, especially on the right side (Table 2).

**Table 2:** Comparison of cerebral nuclei volumes between TD, ET, and HC

Feature	TD^a^ (*n* = 69)	ET^b^ (*n* = 71)	HC^c^ (*n* = 80)	Test statistics	*P* value_adj_	Post-hoc test^d^
Pallidum	4041.28 ± 431.14	3775.04 ± 462.91	3763.51 ± 471.75	10.98	<0.001	a > c***, a > b**
L. Pallidum	2049.19 ± 239.42	1899.35 ± 247.02	1898.06 ± 231.29	12.28	<0.001	a > c***, a > b**
R. Pallidum	1992.08 ± 225.34	1875.69 ± 239.46	1865.46 ± 257.89	7.37	0.005	a > c**
B. Thalamus	14337.29 ± 1544.54	13330.65 ± 1913.97	14007.49 ± 1300.8	10.12	<0.001	a > b**
L. Thalamus	7222.82 ± 781.16	6759.68 ± 1002.81	7004.12 ± 699.08	7.74	0.005	a > b*
R. Thalamus	7114.47 ± 824.69	6570.97 ± 947.49	7003.37 ± 648.91	11.58	<0.001	a > b**, b < c**
CC. Anterior	894.83 ± 119.98	822.01 ± 154.21	831.38 ± 135.78	6.04	0.013	a > b*, a > c*
CC. Mid Anterior	561.33 ± 148.74	506.86 ± 128.24	504.94 ± 105.96	5.23	0.020	a > c*
B. VentralDC	7690.48 ± 766.3	7265.86 ± 839.84	7537.64 ± 746.14	7.98	0.005	a > b*
L. VentralDC	3852.87 ± 398.57	3641.83 ± 413.55	3787.08 ± 386.48	7.63	0.005	a > b*
R. VentralDC	3837.61 ± 393.83	3624.03 ± 442.7	3750.57 ± 377.24	7.22	0.005	a > b*
WM. hypointensities	2604.1 ± 2553.11	3175.72 ± 4371.85	1463.77 ± 987.71	7.34	0.005	b > c**
Brain Stem	21416.03 ± 2378.05	20052.45 ± 2645.77	20816.15 ± 2152.8	7.27	0.005	a > b*
Amygdala	1527.89 ± 217.76	1497.29 ± 244.46	1597.2 ± 214.15	5.88	0.014	a < c*
B. Cerebellum Cortex	101448.11 ± 9758.1	98549.25 ± 11812.63	102938.32 ± 9006.98	4.54	0.027	b < c*
Cerebellum Cortex	51305.13 ± 5074.38	49677.06 ± 6036.96	52236.99 ± 4618.99	5.76	0.014	b < c*
B. Inf Lat Vent	1155.31 ± 597.25	1257.03 ± 807.95	956.47 ± 533.16	5.49	0.017	b > c*
R. Inf Lat Vent	558.45 ± 319.44	648.73 ± 471.98	478.95 ± 266.1	5.11	0.020	b > c*
3rd Ventricle	1553.73 ± 529.14	1652.7 ± 627.74	1413.37 ± 529.57	4.93	0.022	b > c*
CSF	1201.75 ± 272.39	1271.48 ± 379.47	1128.54 ± 247.32	4.90	0.022	b > c*
R. Choroid plexus	711.79 ± 190.14	776.23 ± 228.4	683.37 ± 222.47	4.37	0.030	b > c*

Data was shown as mean ± standard error. Analysis of covariance was performed to observe differences among groups. For regional analyses, multiple comparisons across the regions were adjusted by the false discovery rate (FDR) method. Test statistics of F were labeled.

*B* both, *R* right, *L* left, *adj* adjusted.

^a^TD tremor-dominant Parkinson’s disease.

^b^ET Essential tremor.

^c^HC Healthy controls.

^d^Post-hoc pairwise comparisons were adjusted by the Bonferroni method.

*p < 0.05; **p < 0.01; ***p < 0.001.

### SCN analysis of brain regions

**Fig. 2:**
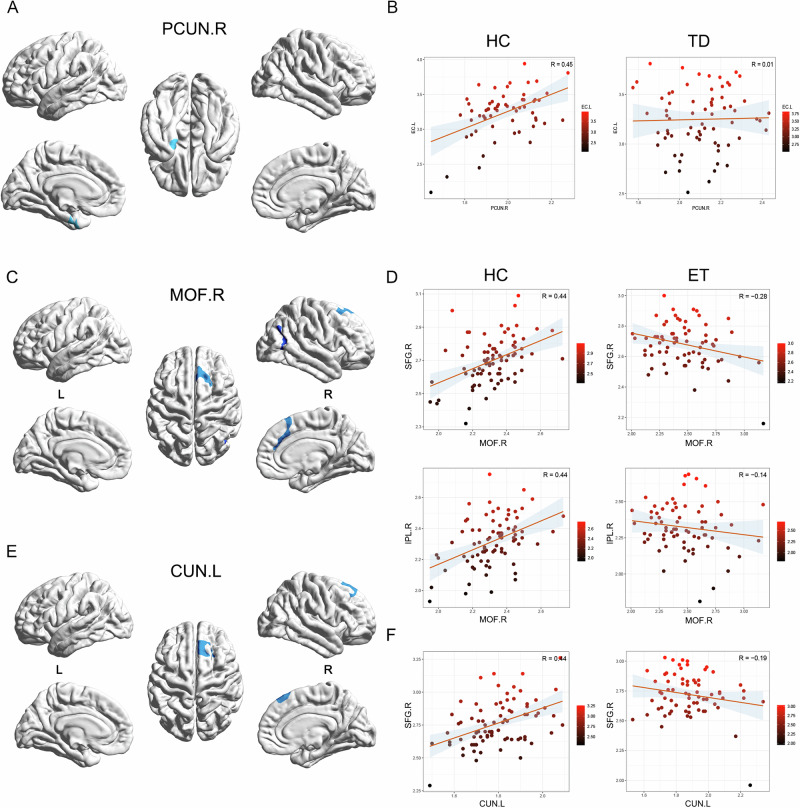
Vertex-SCN results show that the brain regional covariance decreases in TD and ET patients. **A** The covariance decreased brain region with seeding at PCUN.R. **B** Scatter plots visualize the decreased covariance of PCUN.R with EC.L in TD patients. **C** The covariance decreased brain region with seeding at MOF.R. **D** Scatter plots visualize the decreased covariance of MOF.R with SFG.R and IPL.R in ET patients. **E** The covariance decreased brain region with seeding at CUN.L. **F** Scatter plots visualize the decreased covariance of CUN.L and SFG.R in ET patients. All the results have been corrected by GRF method. PCUN.R the right precuneus, EC.L the left entorhinal cortex, CUN.L the left cuneus, MOF.R the right medial orbitofrontal cortex, SFG.R the right superior frontal gyrus, SCN Structural Connectivity Network, GRF Gaussian Random Field correction.

Compared to HC, TD patients showed a thickness covariance decreased between the left entorhinal cortex (EC.L) and PCUN.R (Fig. 2A, B). In ET patients, decreased thickness covariance was observed in the right superior frontal gyrus (SFG.R) and right inferior parietal lobule (IPL.R) with the MOF.R, as well as in the SFG.R with the CUN.L (Fig. 2C–F). No significant increase in thickness covariance was observed between the two patient groups with HC.

### ROIs-based resting-state fMRI analysis

To further explore the specific functions of the ROIs, we performed resting-state fMRI (rs-fMRI) analysis. In TD patients, rs-fMRI revealed decreased the Amplitude of Low-Frequency Fluctuations (ALFF) values in the cerebellar vermis, inferior occipital cortex, bilateral postcentral gyrus, and bilateral precentral gyrus (Supplementary Fig. 2A). Conversely, increased ALFF was observed in the bilateral inferior and middle temporal gyrus and the hippocampus. The PCUN.R-based functional connectivity (FC) analysis demonstrated reduced connectivity in bilateral sensorimotor cortices and parts of the temporal lobe (Supplementary Fig. 2B). These findings suggested that PCUN.R is a core region involved in both structural and functional alterations in TD.

To further investigate the function of the MOF.R in ET patients, we conducted the rs-fMRI analysis. The results revealed a significant decrease in ALFF values in the bilateral thalamus region of ET patients (Supplementary Fig. 3A). Next, we performed ROI-wise FC analysis based on the thalamus and MOF.R. Thalamus-based FC analysis showed that the FC of the thalamus was increased in the bilateral superior frontal gyrus (SFG) in ET patients (Supplementary Fig. 3B), while the MOF.R -based FC analysis showed that the increased FC of MOF.R was also manifested in the bilateral SFG in ET patients (Supplementary Fig. 3C). The Kendall’s Coefficient of Concordance-Regional Homogeneity (KCC-ReHo) analysis revealed that patients with ET enhanced neuronal homogeneity in the frontal region, with voxel signals in MOF being consistent or synchronized with the SFG region (Supplementary Fig. 3D). These results indicated that there were significant FC changes between the thalamus and MOF.R in ET patients.

### Causal effects of patterns in thickness alteration in TD and ET patients

**Fig. 3:**
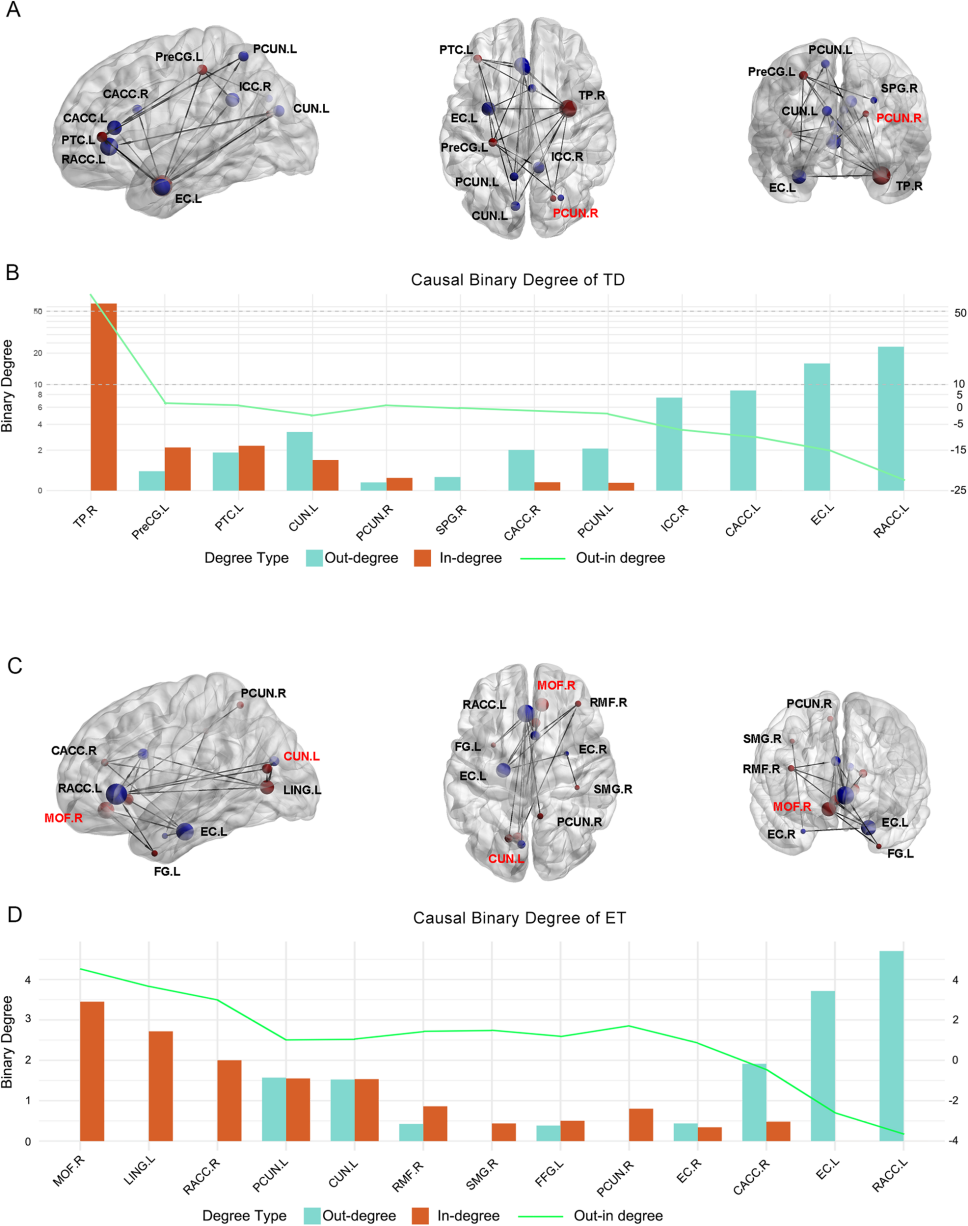
CaSCN analyses show two different structure networks between two groups. **A**, **C** Bivariate signed-path coefficient Granger causality analysis was performed to construct a regional causal network in TD and ET group. **B**, **D** The binary out-(blue) and in-degree (orange) value of each brain region in CaSCN. CaSCN Causal structural covariance networks.

**Fig. 4:**
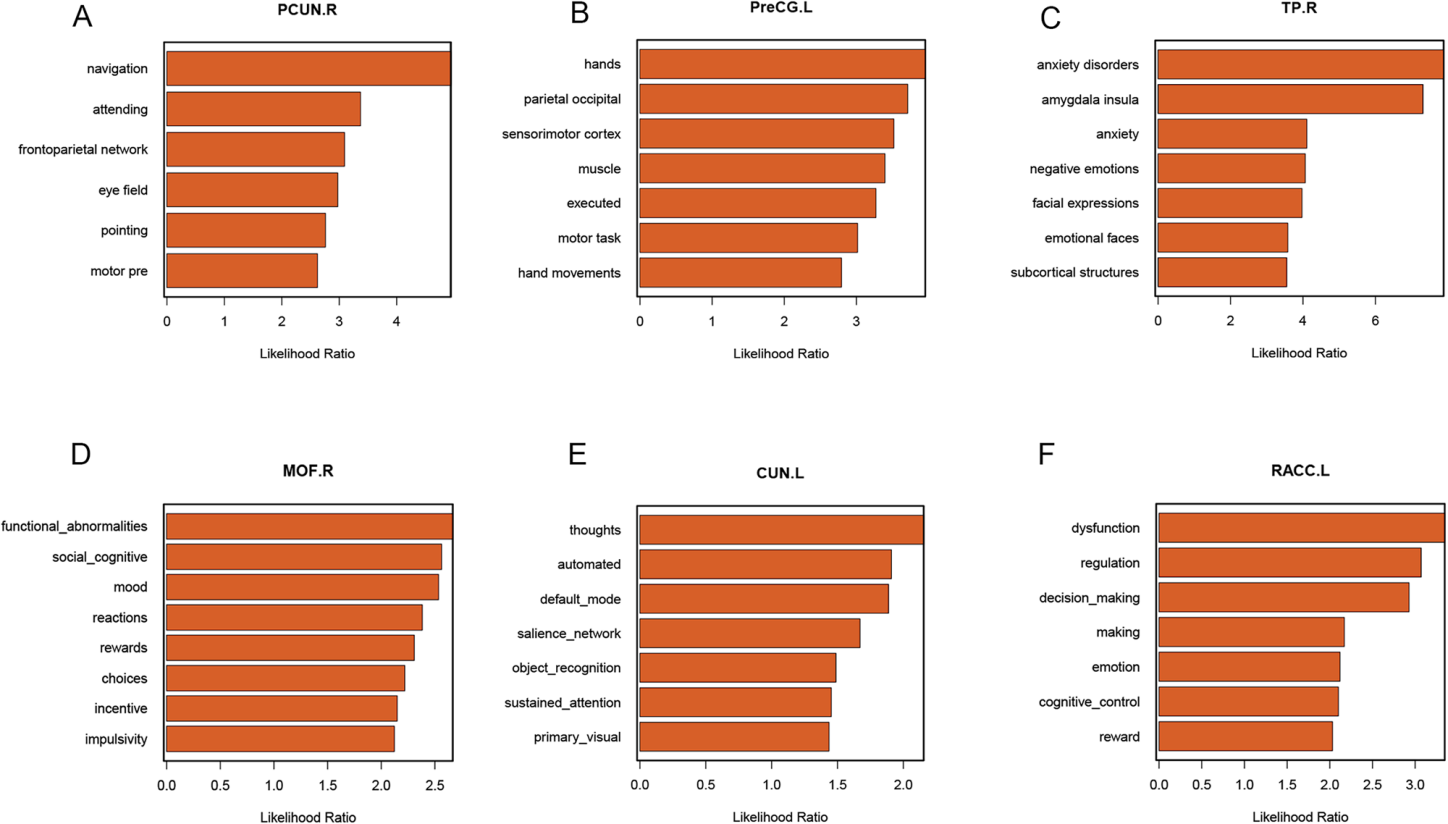
Functional decoding of some high in or out degree regions. **A** Functional decoding of PCUN.R. **B** Functional decoding of PreCG.L. **C** Functional decoding of TP.R. **D** Functional decoding of MOF.R. **E** Functional decoding of CUN.L. **F** Functional decoding of RACC.L. PCUN.R the right precuneus, PreCG.L the left precentral gyrus, TP.R the right temporal pole, MOF.R the right medial orbitofrontal cortex, CUN.L the left cuneus, RACC.L the left rostral anterior cingulate.

In the TD group, only the region PCUN.R exhibited significant changes in SCN, for which it was selected as the seed for the CaSCN analysis. The GCA revealed positive values primarily in the EC.L, left precuneus (PCUN.L), left caudal anterior cingulate (CACC.L), left rostral anterior cingulate (RACC.L), right temporal pole (TP.R), right caudal anterior cingulate (CACC.R), the CUN.L, left parstriangularis (PTC.L), left precentral gyrus (PreCG.L), the SPG.R, and right isthmus cingulate (ICC.R). The ROIs-level CaSCN analysis generated a directed network showing causal relationships between brain regions (Fig. 3A). In this network, TP.R had the highest out-degree, primarily projecting to other nodes, while EC.L and RACC.L had the highest in-degree, receiving causal effects from other nodes (Fig. 3B). Functional decoding was applied to explore the main functions of the brain regions. PCUN.R was primarily associated with navigation, attention, the frontoparietal network, eye field, pointing, and motor planning (Fig. 4A). PreCG.L was mainly related to hand movements, the parieto-occipital region, the sensorimotor cortex, muscle activity, motor execution, and motor tasks (Fig. 4B). TP.R was primarily linked to anxiety disorders, the amygdala, insula, negative emotions, facial expressions, emotional faces, and subcortical structures (Fig. 4C).

In the ET group, MOF.R and CUN.L were selected as the ROIs for the CaSCN analysis. The GCA revealed positive values mainly in the bilateral parietal lobes (PCUN.L, PCUN.R, and right supramarginal gyrus [SMG.R]), occipital lobe (left lingual gyrus [LING.L]), frontal lobe (right rostral middle frontal gyrus [RMF.R]), bilateral temporal lobes (FG.L, EC.L, and right entorhinal cortex [EC.R]), and bilateral cingulate gyrus (right rostral anterior cingulate [RACC.R], the RACC.L, and the CACC.R (Fig. 3C). In this network, MOF.R had the highest out-degree, primarily projecting to other nodes, while the RACC.L had the highest in-degree (Fig. 3D). Functional decoding analysis showed that MOF.R was primarily associated with functional abnormalities, social cognition, mood, reactions, rewards, choices, incentives, and impulsivity (Fig. 4D). CUN.L was primarily related to thoughts, automated, default mode, salience network, object recognition, sustained attention, and primary visual (Fig. 4E). RACC.L was mainly connected to dysfunction in regulation, decision-making, emotion, cognitive control, and reward (Fig. 4F).

### Distinguishing tremor types based on brain morphology and clinical features

**Fig. 5:**
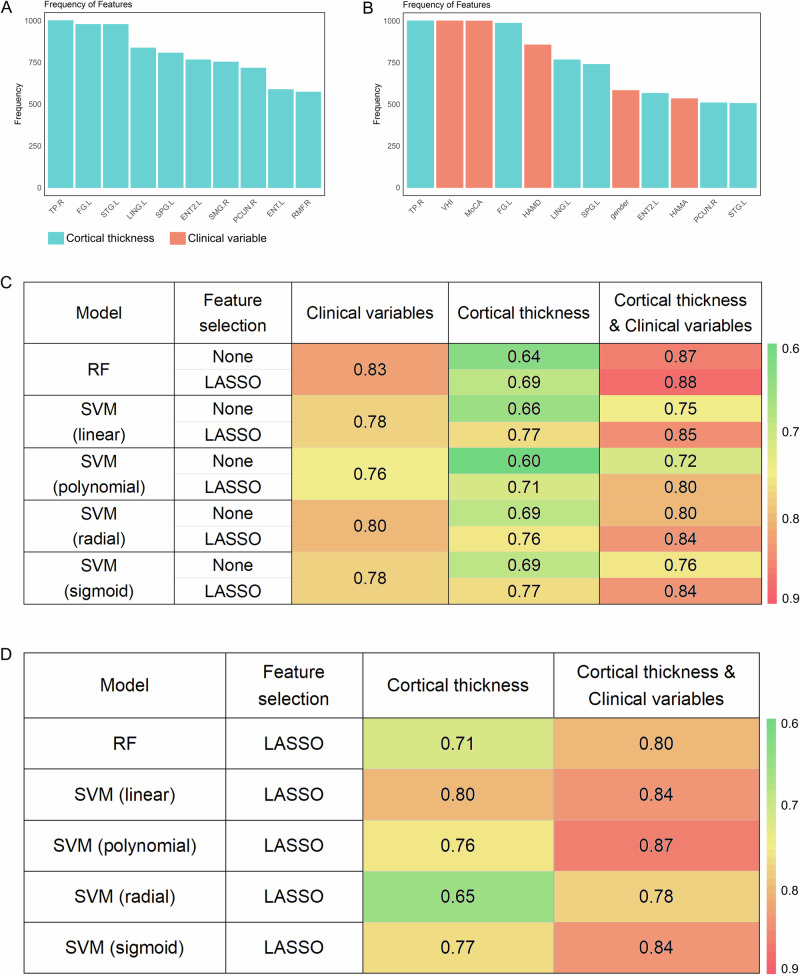
Artificial intelligence classification model based on brain morphology and clinical features. **A** Bar chart showing feature importance ranking for clinical variables. **B** Bar chart showing feature importance ranking for brain morphology and clinical variables. **C** Heatmap of AUC value from various machine learning models for predicting tremor type in test set. **D** The performance on external test set. RF random forest, SVM support vector machines, LASSO least absolute shrinkage and selection operator.

Machine learning was employed to distinguish different tremors based on cortical thickness measurements. The least absolute shrinkage and selection operator (LASSO) algorithm is used for feature screening, where we select the first 30% after 1000 operations as important features. In terms of brain morphology, TP.R, FG.L, STG.L, LING.L, SPG.L, ENT2.L, SMG.R, PCUN.R, ENT.L and RMF.R were selected (Fig. 5A). Among the combinations of brain morphology and clinical features, TP.R, VHI, MoCA, FG.L, HAM-D, LING.L, SPG.L, Gender, ENT2.L, HAM-A, PCUN.L and STG.L were selected (Fig. 5B). Figure 5C summarize the model performance on the test cohort. In the test cohort, the model incorporating a combination of brain morphology and clinical variables (the area under the receiver operating characteristic curve [AUC], 0.72–0.87) exhibited superior performance compared to models utilizing solely clinical features (AUC, 0.76–0.83) or brain morphology (AUC, 0.60–0.69). Notably, after adding LASSO feature screening, the prediction performance greatly increased, and fewer variables were included. After using feature filtering, the combined model using brain morphology and clinical variables performed better than other models (AUC, 0.80–0.88). Therefore, we only use models trained with feature selection to validate them in the external test set. In the external test set, models trained using cortical thickness maintained good performance, with AUC values ranging from 0.65 to 0.80. Models incorporating both cortical thickness and clinical parameters demonstrated excellent performance, with AUC values ranging from 0.78 to 0.87 (Fig. 5D).

## Discussion

PD and ET are the two most common diseases cause tremor and also the most prevalent movement disorders. However, as these diseases progress, the emergence of certain atypical subtypes and overlapping symptoms make it increasingly challenging to distinguish between the two conditions based solely on clinical manifestations. In this study, the tremor patients we mainly investigated included TD patients and classic ET patients. Using structural MRI imaging, we screened and constructed the CaSCN brain network to investigate similarities and differences between the two patient groups. Based on these findings, we developed a machine learning model aimed at assisting in the clinical differentiation of the two types of tremors.

In TD patients, results showed an increased cortical thickness in the SPG.L and PCUN.R, and decreased in the MTG.L. In contrast, ET patients demonstrated increased cortical thickness in the MOF.R, the CUN.L, the CUN.R, the FG.L, and the IPG.L, but decreased cortical thickness in SPG.L. These findings were consistent with several previous studies. A study on cortical thickness in PD reported reductions in several brain regions, including the superior frontal gyrus, precentral gyrus, superior temporal gyrus, temporal pole, superior parietal lobule, and precuneus^20^. Another meta-analysis showed that MOF.R and FG.L cortical thickness increased in ET patients^21^. A study showed significant volumetric expansion in the occipital fusiform cortex, right inferior temporal gyrus, and central lobes in ET, which is consistent with our results^22^. Notably, in this study, we found that the cortical thickness near the parieto-occipital sulcus was increased bilaterally in both TD and ET patients. A study on glaucoma suggested that patients might experience compensatory hypertrophy, leading to increased gray matter thickness in the occipital lobe^23^. It is hypothesized that the observed increase in cortical thickness near the parieto-occipital sulcus may result from compensatory hypertrophy associated with reduced motor control in patients with tremors. To determine whether these structural changes emerge in the early disease course, we examined brain morphological differences between early-stage TD and ET patients. Specifically, the early-stage TD patients showed increased cortical thickness in the PCUN.R and the PostCG.R, while the early-stage ET patients demonstrated increased cortical thickness in the MOF.R, STG.R, and SPG.L. These results indicate that brain morphological remodeling is already evident in the early stages of the disease.

Our findings in subcortical structures revealed significant volumetric alterations with the pallidum in TD patients, while in ET patients in the thalamus. These findings further support the hypothesis that PD and ET exhibit distinct pathophysiological mechanisms in subcortical nuclei. The pallidum plays a pivotal role in regulating voluntary motor control and is closely associated with a range of PD symptoms, including bradykinesia and tremors^24^. Studies have demonstrated that alterations in the pallidum may disrupt effective motor signal transmission, potentially resulting in pronounced tremors and other motor impairments^25,26^. Conversely, the thalamus plays a central role in the pathogenesis of ET, particularly in the generation and propagation of tremors. Studies have shown that the ventral intermediate nucleus of the thalamus receives cerebellar input and regulates motor cortical activity through feedback loops, contributing to tremor generation in ET patients^27^.

The SCN analysis characterizes covariant relationships between brain region morphology, reflecting patterns of gray matter loss in neurodegenerative diseases^28^. In TD patients, covariance changes were observed only in the PCUN.R. It is a region widely recognized for its critical role in integrating sensory information to guide motor actions and visual-spatial processing. A study reported that in PD patients, FC between the PCUN and motor-related brain regions is significantly reduced, potentially leading to difficulties in motor planning and execution, manifesting as tremors and rigidity^29^. Another study suggested that repetitive transcranial magnetic stimulation targeting the PCUN region in PD patients with freezing of gait increased excitability in the posterior parietal cortex, resulting in a significant reduction in freezing episodes and freezing time, while also improving visuospatial processing^30^. Consistent with these findings, our rs-fMRI results further support the role of PCUN.R in TD patients. The ALFF analysis revealed decreased spontaneous neural activity in the cerebellar vermis, inferior occipital cortex, bilateral primary somatosensory areas, and bilateral precentral gyri. Meanwhile, PCUN.R-based FC analysis demonstrated reduced connectivity between the PCUN.R and regions surrounding the bilateral central sulcus, including the sensorimotor cortices and parts of the temporal lobe. These findings reinforce the notion that PCUN.R is not only structurally but functionally affected hub in TD, potentially mediating the interaction between non-motor and motor networks.

The SCN result in ET patients demonstrated a significant reduction in the covariance in the MOF.R and the CUN.L. The MOF region has been recognized in numerous studies for its role in integrating information and guiding decision-making, action selection and motor planning, which may influence the manifestation of tremors^31–34^. Studies have shown that the MOF region exhibited increased functional correlations with the dentate nucleus and thalamus in ET patients, suggesting its role in integrating subcortical and cortical information, which aligns with our findings in the subcortical nuclei analysis^35,36^. Further rs-fMRI studies confirmed the connection between MOF and the thalamus in ET patients. The FC analysis of the thalamus revealed increased connectivity in the bilateral SFG, and a similar pattern was observed in the MOF.R-based FC analysis, which also showed enhanced connectivity in the bilateral SFG. Subsequent KCC-ReHo analysis demonstrated the enhanced neuronal homogeneity in the frontal region in ET patients. These findings suggested the MOF established connections with the thalamus through bilateral SFG, highlighting the critical role of MOF in ET.

CaSCN allows the estimation of the causal impact of morphometric alterations in one region and can predict subsequent structural changes in other brain networks associated with disease progression^37^. Comparing the different structural change patterns of morphometric data in these two diseases, provides deeper insights into the pathological progression of this disease. Therefore, we conducted two different seed-based CaSCN analyses based on the duration of TD and ET in patients. In the TD causal network, PCUN.R was selected as the ROI. The result demonstrated that the TP.R exhibited the highest out-degree, primarily influencing the PreCG.L, the PCUN.R, and other brain regions. Studies in PD patients have shown that TP.R is not only associated with motor symptoms but also the cognitive and emotional impairments^38,39^. Li et al. evaluated PD whole brain spontaneous activity through regional homogeneity (ReHo) and reported that patients had increased ReHo in TP.R and decreased ReHo in the primary motor cortex and premotor area^40^. This study further suggests that ReHo in the TP.R is significantly associated with depression and anxiety, indicating that altered brain activity in this region may play a compensatory role in PD. Consistent with this finding, our result in functional decoding of the TP.R indicated that this region was critically involved in anxiety disorders, negative emotions, or facial expressions. In addition, our results showed the PreCG.L serves as the primary downstream hub regulated by TP.R and further projects to the extensive frontal and temporal lobes. Functional decoding of the PreCG.L also indicated that this region was critically involved in execution and motor control. Considering that previous studies have reported that some symptoms (i.e., olfactory dysfunction, rapid eye movement sleep behavior disorder, constipation, and depression) often precede motor symptoms in PD progression, this finding may help explain the temporal pattern of PD progression^41,42^.

In ET patients, the MOF.R and CUN.L were selected as the ROIs. In this causal network, the MOF.R showed the highest out-degree, primarily influencing LING.L, RACC.R, CUN.L and other brain regions. Prasad et al. performed probabilistic tractography in ET patients and reported that global and local efficiency was reduced in regions including the lateral prefrontal cortex, bilateral RACC, and MOF.R^36^. Combined with the previously analyzed SCN and rs-fMRI results, MOF.R has a functional connection with the SPG or thalamus involved in the regulation of cortical activity. These findings suggested that TD and ET may associated with distinct pathophysiological mechanisms of tremors. In our study, TD originated in the temporal lobe and spread to surrounding regions via the motor cortex, whereas ET exhibited initial alterations in the prefrontal lobe, with progression primarily involving the temporal and occipital lobes, but less in the parietal lobe.

By summarizing the different changes in the cortex between TD and ET patients, we may be able to further distinguish these two types of tremors from an imaging perspective. Hence, our objective was to develop an AI model that effectively discriminates between different tremor types individually by utilizing brain morphology as independent features or in combination with clinical information. In the test cohort, models trained using brain morphology variables showed similar performance to models trained using clinical variables. The model performance improved when the model was trained using brain morphology combined with clinical variables rather than using only a single variable. The model’s predictive performance has improved significantly when employing feature filtering with fewer variables. This result was also verified in the external test set. Our results demonstrated that integrating brain morphology and clinical variables into machine learning models significantly enhances the capacity to differentiate between tremor types at an individual level, potentially offering a more refined diagnostic tool for clinical applications.

Despite these important findings, several limitations of our study must be acknowledged. First, the sample size was relatively limited due to the strict diagnostic inclusion and exclusion criteria applied during participant selection. Additionally, the duration of MRI scanning presents a significant challenge for patients with tremors, making it necessary to exclude suboptimal scans to ensure high image quality. Second, the CaSCN analysis was based on a pseudo-time series derived from patients’ disease durations. While this approach reflects the relative strength of causal influence between brain regions, it does not directly capture the actual temporal dynamics of disease progression. Third, although our AI model demonstrated robust performance in both internal and external validation cohorts, the current sample size was insufficient to statistically determine the model’s diagnostic accuracy across different disease stages. In future work, we aim to address these limitations through large-scale, multi-center cohort studies and longitudinal follow-up to further validate and refine our findings.

In conclusion, this article provides a comprehensive study of the structural alterations in the cerebral cortex and subcortical nuclei in tremors, identifying a critical cortical region that exhibits early pathological impairment, offering new insights into the disease progression. Additionally, by incorporating these specific characteristics between those two group patients, we constructed a model that accurately distinguishes between different tremor types at the individual level, offering valuable guidance for clinical practice.

## Methods

### The inclusion criteria for two group patients

The diagnosis of PD was confirmed by multiple physicians following the International Parkinson and Movement Disorder Society Clinical Diagnostic Criteria for Parkinson’s Disease^43^. Among those patients, TD classification was determined by the ratio of the mean tremor score to the mean postural instability/gait difficulty score in the Movement Disorder Society-Sponsored Revision of the Unified Parkinson’s Disease Rating Scale (MDS-UPDRS)^44^. Patients with a ratio of ≥1.15 were classified as TD^45^. For ET patients, the diagnosis is based on the International Consensus Statement on Classification of Tremor^46^. Patients classified as ET‑plus (i.e., those exhibiting gait disturbances, suspicious dystonic postures, or memory impairments) or those presenting with rest tremor (r-ET) were excluded from this study^2,47^. Both the two group need meet the following criteria: (i) should be older than 50 years; (ii) had exhibited cognitive normalcy, as evidenced by a MMSE score ≥ 24; (iii) had no medical implants contraindicated with cerebral MRI; (iv) had excluded if they had an evidence of traumatic brain trauma, stroke, Alzheimer’s disease and epilepsy. In addition to those ratio requirements, TD patients should have effective treatment with levodopa and/or dopamine therapy for more than 6 months. Any patients taking medications that could potentially affect the brain cortex, such as beta-blockers or antianxiety medications, would be excluded. Finally, 69 TD patients, 71 ET patients and 80 HC were recruited between January 2020 to September 2023. Additionally, patients from the First Affiliated Hospital of Zhengzhou University were recruited as an external test set using the same inclusion and exclusion criteria. A total of 11 ET patients (3 males and 8 females) and 13 TD patients (5 males and 8 females) were included. The Ethics Committee of Beijing Tiantan Hospital approved this study (No. KYSQ 2022-389-01-01). In accordance with the Helsinki Declaration, informed consent was obtained from all participants involved in the study.

### Clinical assessments

Demographic and clinical characteristics of the patients, including age, gender, educational background, and disease duration, were obtained through interviews before the MRI scans. TD patients were evaluated using the MDS-UPDRS to quantify tremors and other clinical manifestations. For ET patients, the Essential Tremor Rating Assessment Scale was used to evaluate the disease severity. The HC group was evaluated based on routine physical examination reports and reconfirmed by radiologists. Individuals with tremors noted in the surgical examination section were excluded. The surgical examination was part of the standard general physical assessment conducted by a surgeon, typically including palpation of the thyroid gland and basic inspection of the chest and abdomen to identify overt abnormalities. During this process, the presence or absence of visible tremors was also recorded. Additionally, patients were evaluated using the HAM-A, the HAM-D, the VHI, and the MoCA.

### MRI data acquisition

In this research, the images of TD patient were collected from Beijing Tiantan Hospital, while the ET patients were recruited from The Beijing Tiantan Hospital, The First Affiliated Hospital of Zhengzhou University and The Fujian Union Hospital. Imaging scans were performed using the same machine model and consistent parameter settings. The detailed parameters were as follows: data acquisition was performed using a Siemens Prisma 3.0T scanner. The acquisition parameters for detailed structural images were as follows: TR = 1560 ms, TE = 1.69 ms, matrix size 256 × 256 mm^2^, flip angle = 8, FOV = 240 mm, scanning time = 5 min 53 s, 192 slices, voxel size 1.0 × 1.0 × 1.0 mm^3^. Additionally, the rs-fMRI dataset was obtained from Beijing Tiantan Hospital. The detailed parameters were as follows: data acquisition was performed using a Siemens Prisma 3.0 T scanner. Slice number = 43, matrix size = 64 × 64, FOV = 220 × 220 mm², TR/TE = 2000/30 ms, FA = 90 deg, slice thickness = 3.2 mm, gap = 0, voxel size = 3.4 × 3.4 × 3.2 mm, dummy scan = 0, number of acquisitions = 240, NEX = 1, parallel acceleration = 2.

### Cortical thickness and subcortical volume analysis

All imaging data were processed using the stable version of FreeSurfer (version 7.1.1). Details of the FreeSurfer analysis process were delineated in earlier publications^48^. Briefly, the standard Freesurfer analysis steps include skull stripping, white matter segmentation, cortical reconstruction, smoothing, mapping, cortical thickness measurement and calculation of subcortical nuclei. The cluster Vertex-wise *P* < 0.01 was used to screen for potential clusters of differences. Cluster-wise correction was performed using Monte Carlo Simulation (MCS), and the threshold was set to *P* < 0.05. FreeSurfer was used to extract the volumes of subcortical structures, including thalamus, hippocampus, amygdala, putamen, globus pallidus, caudate and accumbens. The significance level (*P* value) was set at 0.05, and the false discovery rate (FDR) was appropriately adjusted.

To determine whether these structural changes emerged in the early disease course, we examined brain morphological differences between early-stage TD and ET patients. In this study, early-stage TD patients were defined as those with Hoehn and Yahr (H-Y) stage ≤2, and early-stage ET patients were defined as those with a disease duration of less than 10 years^49,50^. Accordingly, TD (*n* = 20, age 63.50 ± 6.37, mean ± SD; sex = 8:10) and ET patients (*n* = 18, age 63.67 ± 7.06, mean ± SD; sex = 14:6) from the original group who met these criteria were selected to form the early-stage subgroups. Each subgroup was compared with matched HC participants. This analysis aimed to determine whether specific cortical thickness changes could be observed at the early stage of the disease.

### SCN analysis

The SCN analysis, targeting the entire brain structure, is a robust and frequently employed technique^28^. It is instrumental in quantifying the connection strengths between cortical regions and elucidating the structural relationships pertaining to the morphological characteristics of various brain areas. In this study, cortical thickness and nuclei volume were used as morphological measures. The SCN analysis was performed in the SurfStat package^51^. The SCN analysis was conducted using Gaussian Random Field correction with the threshold set at *P* < 0.05.

### Rs-fMRI data analysis

To further explore how the structural and functional alterations interact, we performed rs-fMRI in a cohort of TD and ET patients. The rs-fMRI data for these patients were preprocessed using the RESTplus toolbox^52^. The preprocessing steps for the rs-fMRI data were similar to those outlined in related research^52^. Briefly, these steps included removing the first ten time points, slice-timing correction, head motion realignment, normalization, smoothing, and filtering. Slight variations in preprocessing steps were applied depending on the specific metrics being calculated. In the statistical analysis, the ALFF results were corrected using Family-wise Error (FWE) with a significance threshold of *P* < 0.05. The FC values and the KCC-ReHo results were corrected using FDR, with a voxel-level significance threshold of *P* < 0.005 and a cluster-level threshold of *P* < 0.05.

### CaSCN analysis

CaSCN analysis utilizes the construction of pseudo-time series morphometric data to estimate the causal impact of one brain region on other regions^15,16^. Consistent with the analysis process of CaSCN calculation in previous studies, we constructed a seed-based CaSCN, and the seed regions were obtained from the previously mentioned ROIs and SCN analysis^17^. Specifically, we first ordered the cortical thickness data of all patients by disease duration to generate a pseudo-time series, simulating the temporal progression of cortical remodeling across individuals. Subsequently, signed-path coefficient GCA was performed in a vertex-wise manner across the entire brain. Only positive GC values were retained, and the resulting maps were transformed into z-scores. A statistical threshold of corrected *P* < 0.05 and a cluster size >10 vertices. For ROI-wise CaSCN analysis, we extracted ROIs from the vertex-wise GC maps and computed ROI-to-ROI directional GC values to construct a causal network matrix. To further characterize the causal role of each ROI, binary in-degree (number of incoming connections) and out-degree (number of outgoing connections) values were calculated to identify candidate source and target nodes within the cortical progression network.

### Functional decoding

Functional decoding refers to the method of predicting the functions of ROIs using large-scale meta-analysis databases^53^. We utilized forward inferences to derive the behavioral domain of the task using the BrainMap database^54^. In the process of forward inferences, the functional attributes of ROIs are determined by identifying specific classification labels (domains or subdomains) where the likelihood of activation significantly surpasses the overall probability observed throughout the entire database. Significance was determined using the binomial test at a significance level of *P* < 0.05 and adjusted for multiple comparisons using the FDR correct.

### Feature selection and multi-machine learning

Enrolled patients were randomly divided into a train set and a test set using a 3:1 ratio. Machine learning models are trained in the train set to predict the predictive performance of the model using different feature combinations, machine learning methods, and the presence or absence of feature selection. Feature selection was performed using LASSO regression from the “glmnet” package in R, with 10-fold cross-validation. LASSO regression was repeated 1000 times in the training set and features greater than 500 times were selected to train the machine learning model. This study used five machine learning methods: random forests and support vector machines (linear, polynomial, radial basis function, and sigmoid). For each model, 10-fold cross-validation was performed within the training set to optimize model parameters and assess internal stability. Each machine learning model generates a predicted probability for each patient in the test set at each iteration. The predicted probabilities were retested on 1000 resamples in the train and test cohorts. Predicted probabilities were used to calculate the AUC for each model.

## Supplementary information

Supplementary Information

## Data Availability

Due to data-sharing, data-use or privacy agreements, data cannot be made available. Analytic code can be made available upon request to the corresponding author.
