# Supplementary material for: Unraveling morphological brain network disparities Parkinsonian tremor from essential tremor: an artificial intelligence approach for clinical differentiation
Source: NPJ Parkinsons Dis. 2025 Aug 22;11:253. doi: 10.1038/s41531-025-01107-8 (PMC12373744; doi:10.1038/s41531-025-01107-8)
Supplement: Supplementary file 1 — Supplementary Information [file 41531_2025_1107_MOESM1_ESM.pdf]

Supplement to: Unraveling Morphological Brain Network Disparities Parkinsonian Tremor from Essential Tremor: An Artificial Intelligence Approach for Clinical Differentiation

Moxuan Zhang[1,2,7], Siyu Zhou[1,2,7], Huizhi Wang[1,2], Pengda Yang[1,2], Jinli Ding[3], Xiaobo Wang[3], Xuzhu Chen[3], Chaonan Zhang[1,2], Anni Wang[1,2], Yuan Gao[1,2], Qiang Liu[1,2], Yueping Li[4], Tianqi Xu[5], Zeyu Ma[5], Yin Jiang[1], Lin Shi[2], Chunlei Han[2], Yuchen Ji[5✉], Guoen Cai[4✉], Tao Feng[6✉], Jianguo Zhang[2✉], Fangang Meng[1,2,5✉]

[1]Beijing Neurosurgical Institute, Capital Medical University, 100070 Beijing, China. [2]Department of Neurosurgery, Beijing Tiantan Hospital, Capital Medical University, 100070 Beijing, China. [3]Department of Radiology, Beijing Tiantan Hospital, Capital Medical University, 100070 Beijing, China. [4]Department of Neurology, Fujian Medical University Union Hospital, 350001 Fujian, China. [5]Department of Neurosurgery, The First Affiliated Hospital of Zhengzhou University, 450000 Henan, China. [6]Department of Neurology, Beijing Tiantan Hospital, Capital Medical University, 100070 Beijing, China. [7]These authors contributed equally: Moxuan Zhang, Siyu Zhou. [✉]email: lyjychen@126.com; cgessmu@fjmu.edu.cn; bxbkyjs@sina.com; zjguo73@126.com; fgmeng@ccmu.edu.cn

Corresponding Author:
Fangang Meng, Department of Neurosurgery, Beijing Neurosurgical Institute, Capital Medical University, Beijing 100070, China.
E-mail: fgmeng@ccmu.edu.cn
Jianguo Zhang, Department of Neurosurgery, Beijing Tiantan Hospital, Capital Medical University, Beijing 100070, China.
E-mail: zjguo73@126.com
Tao Feng, Department of Neurology, Beijing Tiantan Hospital, Capital Medical University, Beijing 100070, China.
Guoen Cai, Department of Neurology, Fujian Medical University Union Hospital, Fujian, China.
E-mail: cgessmu@fjmu.edu.cn
Yuchen Ji, Department of Neurosurgery, The First Affiliated Hospital of Zhengzhou University, Henan, China.
E-mail: lyjychen@126.com

Supplementary Fig. 1 The alteration of cortical thickness in early-stage TD and ET patients compared to HC.

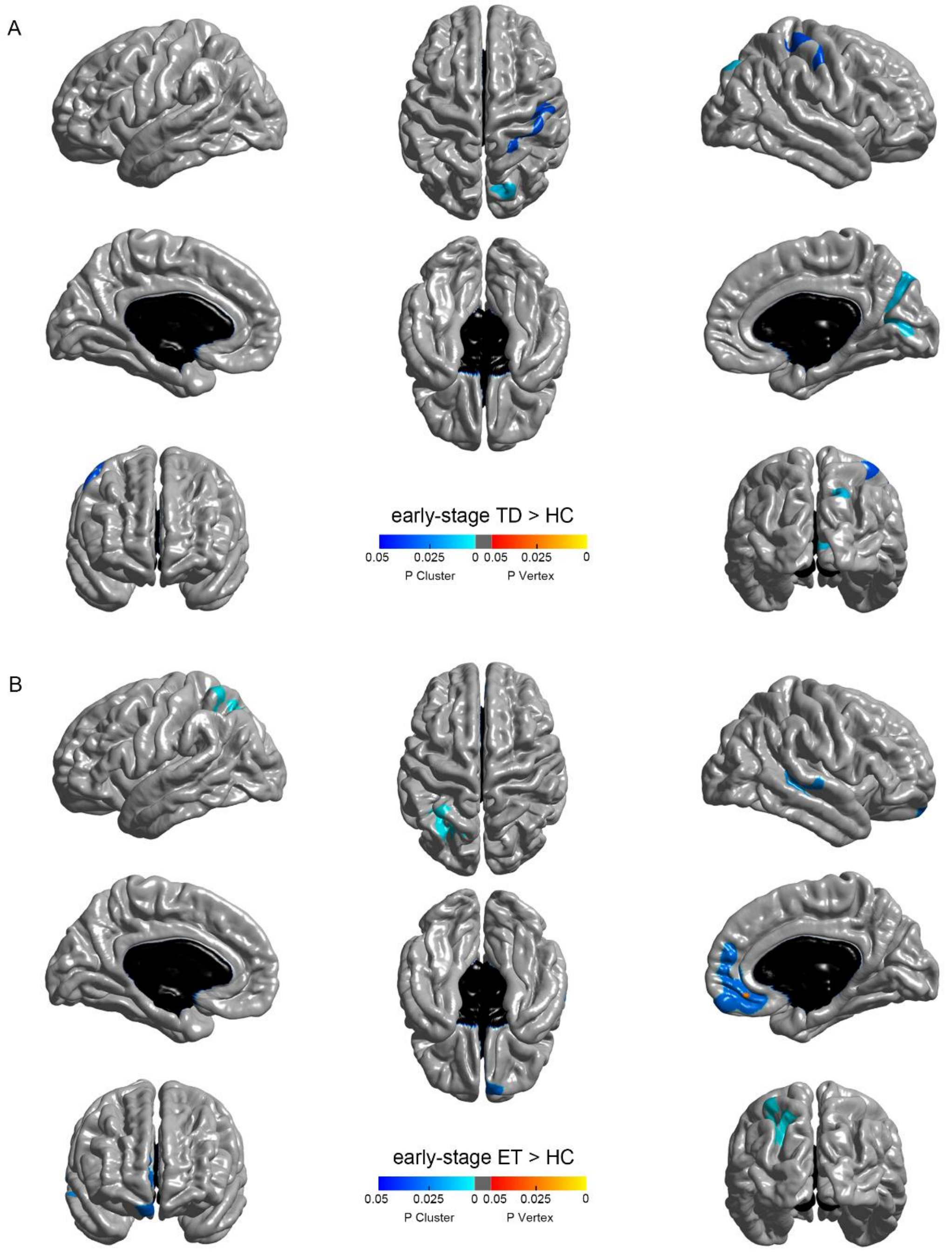

A and **B** Brain regions showing significant cortical thickness increases in early-stage TD and early-stage ET patients compared to HC, visualized on pial brain surfaces by FreeSurfer in MATLAB vision. Colored areas represent increased cortical thickness with varying levels of statistical significance. No regions with cortical thinning were observed in either group. The results were corrected using Monte Carlo Simulation (MCS) with $P < 0.05$. TD Tremor-dominant Parkinson's disease, ET essential tremor, HC healthy controls.

Supplementary Fig. 2 Analysis of rs-fMRI functional alterations in TD and HC.

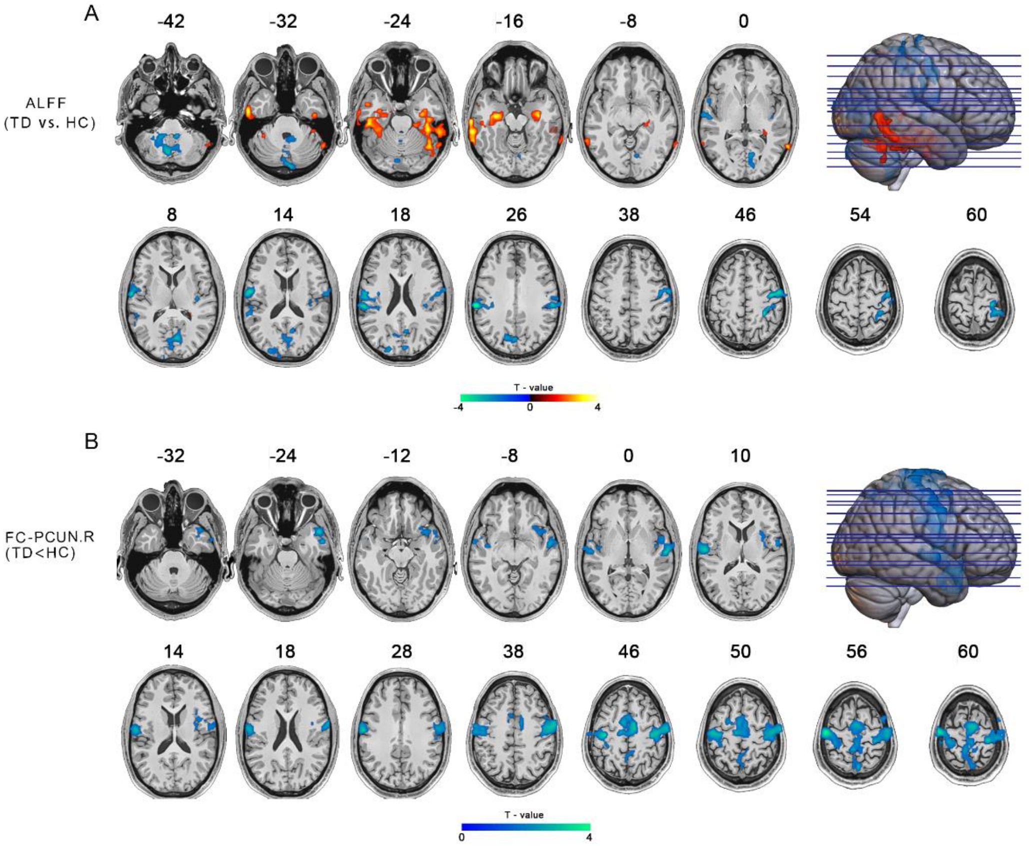

A The results showed significant alterations in ALFF values across multiple regions in TD patients compared with HC. Red areas indicate increased ALFF values, while blue areas indicate decreased ALFF values. **B** FC analysis with the PCUN.R as a seed revealed decreased connectivity with the bilateral sensorimotor cortex and parts of the temporal lobe. TD tremor-dominant Parkinson's disease, HC healthy controls, ALFF the Amplitude of Low-Frequency Fluctuations, FC the functional connectivity.

Supplementary Fig. 3 Analysis of rs-fMRI functional alterations in ET and HC.

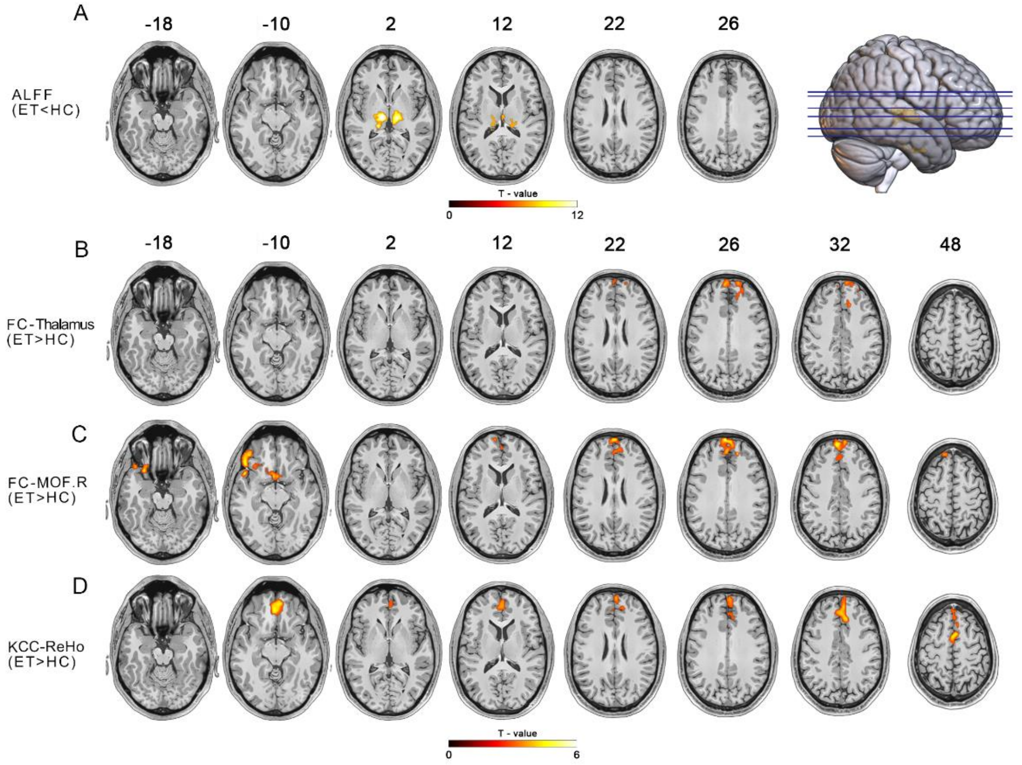

A The results showed that the ALFF value in the bilateral thalamus area was significantly decreased in ET patients. **B** FC analysis with the thalamus as a seed showed enhanced connectivity with the prefrontal cortex. **C** FC analysis with the MOF.R as a seed showed enhanced connectivity with the prefrontal cortex in same region. **D** The KCC-ReHo results indicate that the voxel signals in bilateral MOF are consistent or synchronized with those part in the prefrontal cortex. ET essential tremor, HC healthy controls, ALFF the Amplitude of Low-Frequency Fluctuations, FC the functional connectivity, KCC-ReHo Kendall's Coefficient of Concordance - Regional Homogeneity.

Supplementary Table 1. Significant cortical thickness differences in TD compared to HC from vertex-wise analysis.

Anatomical region	Abbreviations	Talairach coordinates			Max	Cluster size (mm^2)	No. of Vertices	P value
		X	Y	Z				
Left brain								
Superiorparietal.L	SPG.L	-28.4	-55.0	53.2	3.94	801.07	1661	<0.001
Middletemporal.L	MTG.L	-59.2	-14.3	-23.6	-3.43	257.41	401	0.037
Right brain								
Precuneus.R	PCUN.R	20.3	-72.5	20.8	3.69	520.13	790	<0.001

TD Tremor-dominant Parkinson's disease, HC healthy control, Max the peak P value.

Note: The *P* value was corrected using the Monte Carlo simulation (MCS) method.

Supplementary Table 2. Significant cortical thickness differences in ET compared to HC from vertex-wise analysis.

Anatomical region	Abbreviations	Talairach coordinates			Max	Cluster size (mm^2)	No. of Vertices	P value
		X	Y	Z				
Left brain								
Inferiorparietal.L	IPG.L	-35.7	-64.2	41.1	4.32	483.80	1126	<0.001
Superiortemporal.L	STG.L	-42.4	-4.4	-20.5	-3.99	385.85	956	0.003
Cuneus.L	CUN.L	-16.3	-71.6	15.8	5.25	370.66	624	0.004
Fusiform.L	FG.L	-38.4	-45.1	-21.7	3.94	343.22	636	0.007
Right brain								
Cuneus.R	CUN.R	19.3	-70.6	16.5	3.52	535.72	698	<0.001
Medialorbitofrontal.R	MOF.R	9.4	36.3	-12.1	4.99	372.09	649	0.003

ET essential tremor, HC healthy control, Max the peak P value.

Note: The *P* value was corrected using the Monte Carlo simulation (MCS) method.
